# Prediction of difficulty in direct laryngoscopy

**DOI:** 10.1038/s41598-022-13523-4

**Published:** 2022-06-24

**Authors:** Ines Kharrat, Imen Achour, Jihene Jdidi Trabelsi, Majdi Trigui, Wadii Thabet, Malek Mnejja, Bouthaina Hammami, Amine Chakroun, Ilhem Charfeddine

**Affiliations:** 1 Department of Otorhinolaryngology, Habib Bourguiba Hospital, Sfax, Tunisia; 2 Department of Epidemiology and Preventive Medicine, Hedi Chaker Hospital, Sfax, Tunisia; 3 University of Sfax, Sfax, Tunisia; 4 Department of Otorhinolaryngology, Habib Bourguiba Hospital, Sfax, Tunisia

**Keywords:** Clinical trial design, Diseases

## Abstract

To establish easily measurable and reproducible preoperative parameters predicting difficult laryngeal exposure in direct laryngoscopy. A prospective study including 71 patients who underwent transoral microsurgery for benign or malignant lesions of the larynx was performed in our department from January 2021 to November 2021. Physical assessment included the Mallampati score, weight, height, body mass index and measurements of seven parameters in the cervical region. Eleven parameters were measured on the cervical radiography film. Among our patients, 19 were included in the difficult laryngeal exposure (DLE) group. High Mallampati and Cormack scores were significantly associated with DLE (p = 0.005 and p < 0.0001). Limited mouth opening, direct thyromental distance (DTMD) < 67 mm in neutral position, DTMD < 82 mm and sternomental distance < 157 mm at full head extension were statistically related to DLE. For radiological assessment, the effective length of the maxilla and the atlanto-occipital distance were related to DLE. Using stepwise logistic regression, only the effective length of the maxilla and atlanto-occipital distance were selected as independent predictors for DLE (p: 0.015 and 0.001). Preoperative prediction of DLE is useful for both surgeons and patients. The length of the maxilla and the atlanto-occipital distance were found to be independent risk factors for DLE. This highlights the effect of overgrowth of the maxilla, protrusion of the upper teeth and limited extension of the cervical spine as the major risk factors for difficult laryngeal exposure.

## Introduction

Suspension direct laryngoscopy (SDL) is a common procedure in otolaryngology surgery. It has become the gold standard for diagnosing benign and malignant lesions of the larynx or hypopharynx^1^. It is commonly used for the diagnosis and treatment of voice disorders with phonomicrosurgery. This type of procedure promotes the magnification of the image and the possibility of visualizing the endolaryngeal anatomical structures. However, by using rigid laryngoscopes transorally, the view is exclusively frontal in a straight line, which emphasizes the importance of good exposure of the laryngeal structures to properly assess laryngeal lesions and for effective transoral surgery^2^.

SDL is usually a simple procedure without complications^3^. In most cases, the placement of a suspension laryngoscope transorally achieves good exposure of the glottis, including the anterior commissure, which is the most difficult to adequately visualize^3^.

However, in some cases, the surgeon cannot complete the procedure because of difficult laryngeal exposure, which prevents him or her from seeing the anterior part of the glottis, especially the anterior commissure. This situation might lead to misdiagnosis, unnecessary trauma, incomplete surgery and even abortion of the procedure^3^.

In these cases, the sniffing position^4^, external pressure on the cricoid cartilage^5^ and the use of a smaller rigid laryngoscope^6^ could help the surgeon ameliorate the obstructed view and see the totality of the vocal cord. However, in some cases, the visibility of the anterior part remains impossible despite all efforts, which makes the achievement of surgical intervention impossible.

There are relatively few studies that have investigated parameters of difficult laryngeal exposure for SDL because many of them were conducted focusing on the feasibility of endotracheal intubation. The results have been variable and not reproducible. Although impossible exposure of the larynx for SDL is rare in clinical practice, difficult laryngeal exposure during endotracheal intubation and laryngeal microsurgery (LMS) has been determined to occur in 6–27% of patients^7^.

However, physical examination could alert clinicians to potential problems in access to the larynx. For instance, clinical findings such as micrognathia and mandibular anteriorization, as well as measures of the cervical circumference and modified Mallampati index, are known for predicting the risk of difficult airway exposure^8,9^. There is no consensus to predict this difficulty in direct laryngoscopy, and there is no score that allows the preoperative identification of patients who would present a high risk of difficult exposure during direct laryngoscopy. Unfortunately, it is not uncommon that cases with difficult exposure of the larynx during SDL do not reveal themselves until the patient is intraoperative and under general anesthesia, which can be troublesome for the surgeon.

Thus, this study was performed to establish easily measurable and reproducible preoperative parameters, which would be associated with a significant risk of encountering a difficult laryngeal exposure during direct laryngoscopy to allow the surgeon to prepare technically before the start of the procedure and inform the patient about the risks.

We believe that we can establish clinical and/or radiological parameters to predict difficulty in direct laryngoscopy. We also think that radiological parameters may be easily reproducible and more reliable.

## Materials and methods

### Patient selection

A prospective study including 71 consecutive patients who underwent transoral microsurgery for benign or malignant lesions of the larynx was performed in our department from January 2021 to November 2021. The local ethics committee of our hospital (Habib Bourguiba Hospital, Sfax, Tunisia) approved the consent procedure and the whole trial under number 07/21. The study was performed in accordance with the Declaration of Helsinki.

Patients who previously underwent neck surgery, tracheostomy, or radiotherapy for the head and neck and patients who did not consent were excluded from the study.

### Physical examination and measurements

The preoperative evaluation of patients included a clinical evaluation of age, sex, medical and surgical history, the indication for direct laryngoscopy, and general physical measurements that included the Mallampati score, weight, height and body mass index (BMI).

**Figure 1:**
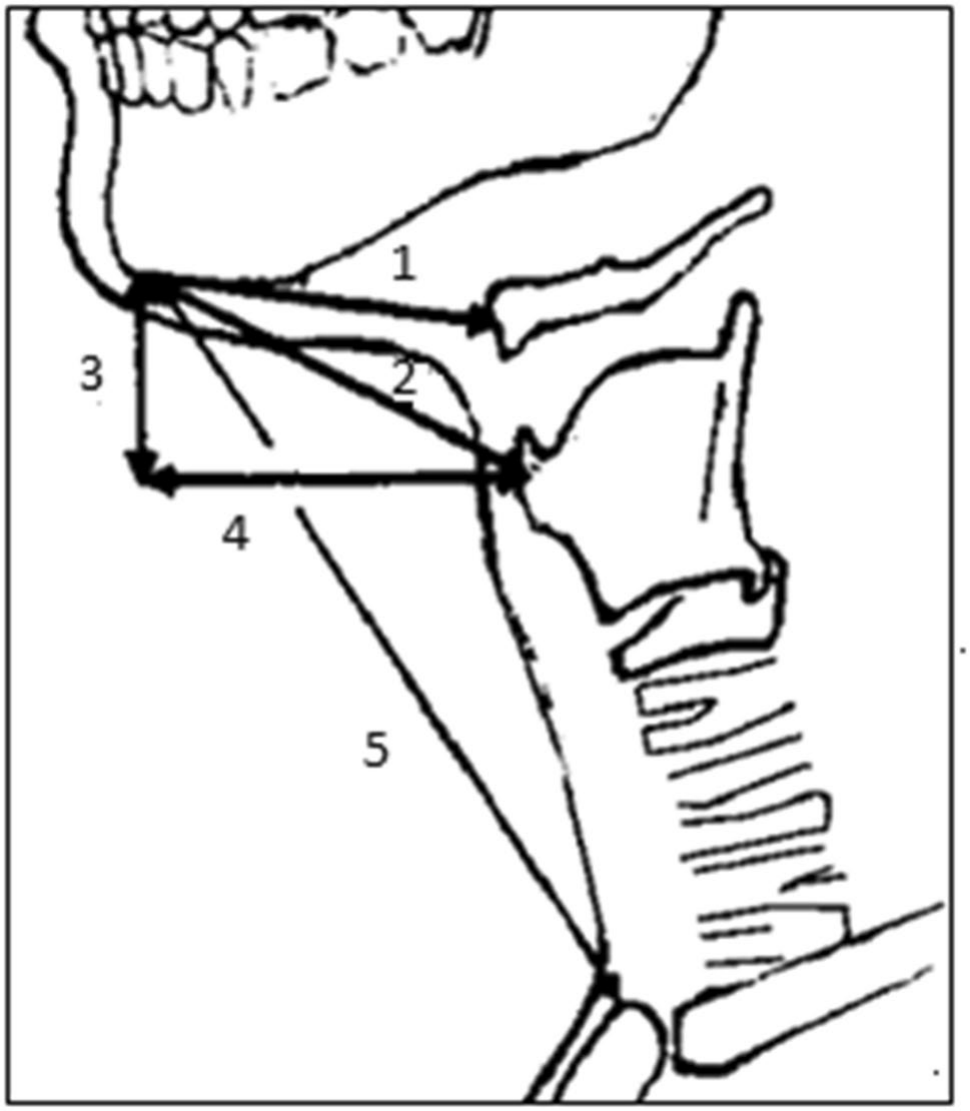
Physical measurements in the neutral position. 1: Hyomental distance (HMD): Distance between the chin and the hyoid bone. 2: Direct thyromental distance (DTMD): Distance between the chin and the thyroid cartilage. 3: Vertical thyromental distance (VTMD): Vertical distance between the chin and the thyroid cartilage. 4: Horizontal thyromental distance (HTMD): Horizontal distance between the chin and the thyroid cartilage. 5: Sternomental distance (SMD): Distance between the chin and the sternum.

Next, all patients underwent different measurements of the cervical region, including seven parameters: neck circumference, mouth opening, hyomental distance (HMD), direct thyromental distance (DTMD), horizontal thyromental distance (HTMD), vertical thyromental distance (VTMD) and sternomental distance (SMD). All these measurements were performed with a standard measuring tape and with the patient in a natural position at the end of expiration and without swallowing (Fig. 1)^8^. The HMD, DTMD, HTMD, VTMD and SMD were then assessed with the head in full extension^8^.

Patients were hospitalized 24 h before the surgical procedure. Each had a standard lateral cervical X-ray: the mouth was fully opened with extension of the head. We used digital radiography that allowed us to obtain radiographic images in real dimensions. We used the same X-ray machine and respected the same parameters and distance between the patient and the device in all cases.

**Figure 2:**
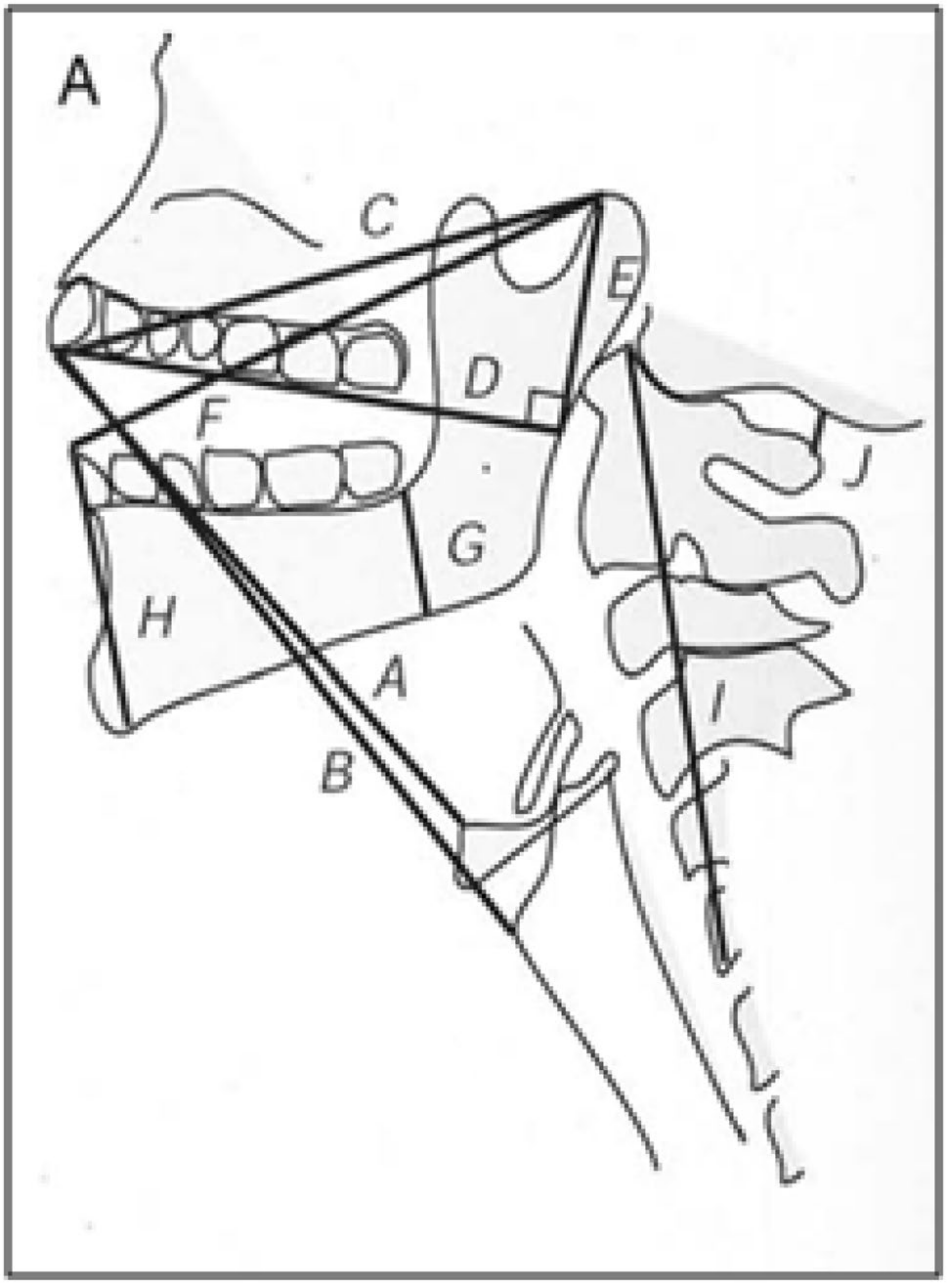
Radiographic measurements. A: Distance between the tip of the upper incisors and the upper border of the body of the hyoid bone. B: Distance between the tip of the upper incisors and the thyroepiglottic junction. C: Distance between the tip of the upper incisors and the temporomandibular joint (effective maxilla length). D: Distance between (along the upper teeth) the tip of the incisors and the point where the line intersects a perpendicular line extending from the temporomandibular joint. E: Distance between (perpendicular line) the temporomandibular joint and Line D (effective height of maxilla). F: Distance between the tip of the lower incisors and the temporomandibular joint. G: Distance between the tip of the alveolus immediately behind the third molar tooth and the lower border of the mandible (posterior depth of the mandible). H: Distance between the tip of the lower incisors and the anterior limit of the mandible’s lower border (anterior depth of the mandible). I: Distance between the upper border of the C1 body and the lower border of the C5 body. J: Distance between the occiput and the spine of C1 (at maximum head extension) (atlanto-occipital distance).

**Figure 3:**
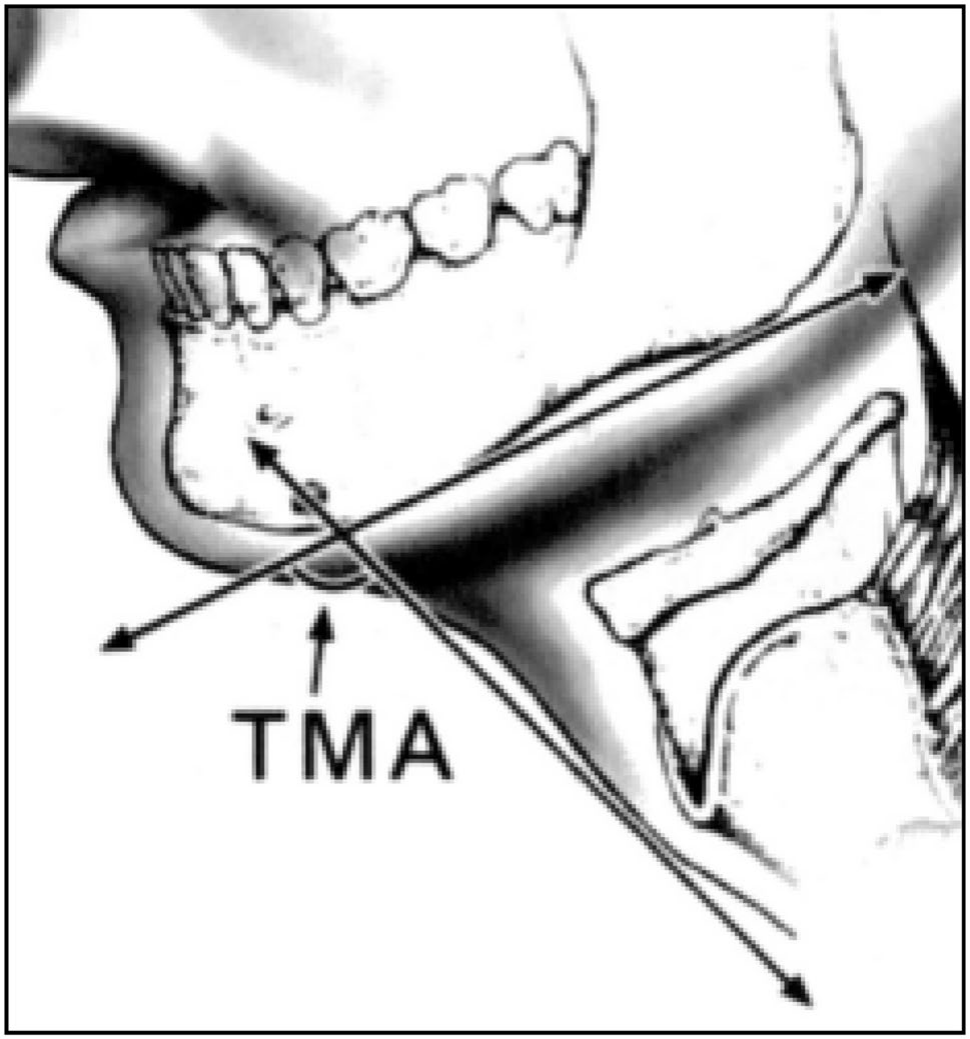
Tyro-mandibular angle^10^. TMA: thyroid-mandible angle: the angle between the line of the mandibular angle to the prominence and the line from the thyroid notch to the mandible.

Eleven parameters were measured on the cervical radiography film (Fig. 2)^10^:

- A: Distance between the tip of the upper incisors and the upper border of the body of the hyoid bone
- B: Distance between the tip of the upper incisors and the thyroepiglottic junction.
- C: Distance between the tip of the upper incisors and the temporomandibular joint (effective maxillary length).
- D: Distance between (along the upper teeth) the tip of the incisors and the point where the line intersects a perpendicular line extending from the temporomandibular joint.
- E: Distance between (perpendicular line) the temporomandibular joint and Line D (effective height of maxilla).
- F: Distance between the tip of the lower incisors and the temporomandibular joint.
- G: Distance between the tip of the alveolus immediately behind the third molar tooth and the lower border of the mandible (posterior depth of mandible).
- H: Distance between the tip of the lower incisors and the anterior limit of the mandible’s lower border (anterior depth of mandible).
- I: Distance between the upper border of the C1 body and the lower border of the C5 body.
- J: Distance between the occiput and the spine of C1 (at maximum head extension) (atlanto-occipital distance).
- TMA: thyroid-mandible angle: the angle between the line of the mandible angle to the prominence and the line from the thyroid notch to the mandible (Fig. 3).

All measurements were conducted by the same doctor using the same method of measurement and respecting the same landmarks described.

### Surgical procedure

Under general anesthesia and muscle relaxation, assessment of the laryngeal view is first performed by anesthesiologists with a Mackintosh laryngoscope blade 3 or 4 and classified according to the Cormack–Lehane score (Table 1)^11^. The patient is intubated with the smallest possible endotracheal tube that allows effective ventilation.

**Table 1:** Cormack–Lehane score.

Grade 1	Full view of the vocal folds
Grade 2	Partial view of the vocal folds or posterior commissure
Grade 3	Only the epiglottis visible
Grade 4	Neither the epiglottis nor the glottis seen

The patient is then put in the sniffing position (with a pillow under the head rendering the neck flexed 35 degrees on the torso and the head extended at the atlantooccipital joint to produce a 15° angle between the facial plane and the horizontal). The surgeon placed at the head of the patient introduces a transoral rigid Kleinsasser laryngoscope. The laryngoscope is passed along the tongue and the tongue base; then, it elevates the epiglottis to allow visualization of the glottis. The laryngoscope holder and the chest device are fixed in the position of the maximum laryngeal view.

In this study, if the vocal cords were not completely seen, the surgeon or his or her assistant exerted a counter pressure on the cricoid and the lower part of the thyroid cartilage to improve vision. If the anterior part of the vocal cord was not yet seen, the laryngoscope was replaced by another, smaller one to allow better vision. If, despite all these efforts, laryngeal exposure was limited to the posterior third of the vocal cords or less, direct laryngoscopy was defined as difficult, and the patient was classified into the difficult laryngeal exposure (DLE) group.

The procedure was not performed on all patients by the same surgeon. However, for all patients, surgeons were assisted by the same surgical assistant, who was responsible for the patient’s position and the instruments we used during the procedure. One surgeon, among the authors, oversaw the procedure in all cases to ensure the respect of all steps.

### Statistical analysis

Statistical analyses were performed using SPSS 23.0 software. We used the X^2^ test to compare the laryngeal exposure scores of the male and female groups. Spearman's p correlation coefficient was used to evaluate the correlation between the parameters and the laryngeal exposure score and the Cormack–Lehane and laryngeal exposure scores. The cutoff points of parameters were defined from the best values obtained from receiver operating characteristic curve analysis. The X^2^ test was used to analyze the associations between the candidate predictors and DLE based on the defined cutoff values.

The ‘t’ test was used to compare continuous variables, and the X^2^ test was used to assess the association between categorical variables.

A multivariable logistic regression model was used to assess predictors that were significantly associated with the outcome at the 10% significance level. Based on diagnostic test criteria (sensitivity, specificity and ROC curve), continuous predictors were dichotomized.

Statistical significance was assumed for p < 0.05.

### Ethics approval

The local ethics committee of our hospital (Habib Bourguiba Hospital, Sfax, Tunisia) approved the consent procedure and the whole trial under number 07/21.

### Consent to participate

Informed consent was obtained from the patients.

## Results

This study included 71 patients who underwent direct laryngoscopy under general anesthesia for benign or malignant lesions of the larynx. There were 59 men and 12 women, and the M:F sex ratio was 4.9. The ages of the patients ranged from 22 to 80 years (with a mean age of 54.9 years and a median of 58 years).

Our patients were divided into two groups based on whether laryngeal exposure was difficult. We noted 19 cases included in the DLE group (26.8%) versus 52 cases included in the other group.

High Mallampati and Cormack scores (grades 3 and 4) were significantly associated with the DLE group (Table 2).

**Table 2:** Correlation of Mallampati and Cormack scores with difficult laryngeal exposure.

Score	Value	Control group	DLE group	P
Mallampati	Class 1 and 2	38	7	0.005
	Class 3 and 4	14	12	
Cormack	Grade 1 and 2	49	3	< 0.0001
	Grade 3 and 4	3	16	

*DLE* difficult laryngeal exposure, *P* Spearman's p correlation coefficient.

The study’s results of the correlation between different parameters (physical and on X-ray film measures) and DLE are summarized in Table 3.

**Table 3:** Correlation of general, physical and X-ray film measures with difficult laryngeal exposure.

Parameter	Minimum	Maximum	Median	P
Age (years)	22	80	58.00	0.608
Weight (kg)	45	106	70.00	0.580
Height (cm)	150	190	167.00	0.850
BMI (kg/m^2^)	16,84	36,35	25.95	0.938
Neck circumference (mm)	300	500	400.00	0.891
Mouth opening (mm)	25	80	45.00	0.05
HMD in neutral position (mm)	30	80	50.00	0.921
DTMD in neutral position (mm)	40	130	70.00	0.05
VTMD in neutral position (mm)	4	70	45.00	0.989
HTMD in neutral position (mm)	25	100	60.00	0.514
SMD in neutral position (mm)	90	170	130.00	0.087
HMD in full extension (mm)	35	98	55.00	0.777
DTMD in full extension (mm)	65	140	90.00	0.021
VTMD in full extension (mm)	40	120	70.00	0.084
HTMD in full extension (mm)	15	80	40.00	0.084
SMD in full extension (mm)	120	220	165.00	0.011
A (mm)	90	160	115.00	0.514
B (mm)	100	207	150.00	0.359
C (mm)	65	125	100.00	0.05
D (mm)	65	123	100.00	0.375
E (mm)	17	47	31.00	0.09
F (mm)	100	140	112.00	0.332
G (mm)	20	48	30.00	0.397
H (mm)	25	65	50.00	0.238
I (mm)				0.648
J (mm)				< 0.0001
TMA (degrees°)	80	130	100.,00	0.087

*kg* kilogram, *cm* centimeter, *m* meter, *mm* millimeter, *BMI* body mass index, *HMD* hyomental distance, *DTMD* direct thyromental distance, *VTMD* vertical thyromental distance, *HTMD* horizontal thyromental distance,* SMD* sternomental distance, *P* Spearman's p correlation coefficient.

Among the physical measures, mouth opening, DTMD in the neutral position, DTMD and SMD in the full extension position showed significant correlations in patients with DLE (Table 4).

**Table 4:** Physical parameters correlated with difficult laryngeal exposure.

Parameter	Median (mm)		P
	Control group	DLE group	
Mouth opening (mm)	49.00	45.00	0.05
DTMD in neutral position (mm)	72.50	65.00	0.05
DTMD in full extension (mm)	90.00	80.00	0.021
SMD in full extension (mm)	170.00	150.00	0.011

*mm* millimeter, *DTMD* direct thyromental distance, *SMD* sternomental distance, *DLE* difficult laryngeal exposure, *P* Spearman's p correlation coefficient.

Based on the defined cutoff values, associations between the parameters and DLE were analyzed with univariate analysis. Limited mouth opening (< 49 mm), DTMD < 67 mm in the neutral position, DTMD < 82 mm and SMD < 157 mm in full extension of the head were statistically related to DLE (Table 5).

**Table 5:** Univariate analysis of the significant parameters using defined cutoff values.

Measure	Cutoff value	Number of patients		P value
		Control group	DLE group	
Mouth opening	< 49 mm	26	15	0.033
	> 49 mm	26	4	
DTMD neutral position	< 67 mm	13	10	0.028
	> 67 mm	39	9	
DTMD full extension	< 82 mm	15	11	0.024
	> 82 mm	37	8	
SMD full extension	< 157 mm	15	12	0.008
	> 157 mm	37	7	

*DTMD* direct thyromental distance, *SMD* sternomental distance, *mm* millimeter, *DLE* difficult laryngeal exposure, *P* Spearman's p correlation coefficient.

The comparisons between difficult cases and control cases for each of the eleven parameters measured on the cervical radiography film showed a significant difference in two predictors, namely, the effective length of the maxilla (p: 0.05) and the atlanto-occipital distance (p < 0.0001) (Table 6). The univariate analysis showed that an effective length of the maxilla greater than 90 mm and an atlanto-occipital distance less than 6 mm represented significant predictors of DLE. (Table 7).

**Table 6:** Radiographic parameters correlated with difficult laryngeal exposure.

Parameter	Median (mm)		P
	Control group	DLE group	
Effective length of the maxilla (mm)	100.00	106.00	0.05
Atlanto-occipital distance (mm)	6.50	4.00	< 0.0001

*mm* millimeter, *DLE* difficult laryngeal exposure, *P* Spearman's p correlation coefficient.

**Table 7:** Univariate analysis of the effective length of the maxilla and atlanto-occipital distance using defined cutoff values.

Measure	Cutoff value	Number of patients		P value
		Control group	DLE group	
Effective length of the maxilla	> 90 mm	34	18	0.015
	< 90 mm	18	1	
Atlanto-occipital distance	< 6 mm	21	15	0.007
	> 6 mm	31	4	

*mm* millimeter, *DLE* difficult laryngeal exposure, *P* Spearman's p correlation coefficient.

All these factors were included in a multivariable logistic regression model to identify independent predictors. Mallampati and Cormack scores were controlled in the multivariate analysis to avoid bias in as much as the factors leading to high Mallampati or Cormack scores are very intricately related to those leading to DLE. Among all six assessed factors, only the effective length of the maxilla and atlanto-occipital distance were selected by stepwise logistic regression to be independent predictors for DLE (p: 0.015 and 0.001, respectively).

When the HTMD is controlled in full extension, the DTMD in neutral position seems to become correlated with DLE (p: 0.049).

## Discussion

Difficult laryngeal exposure, although not a common situation encountered in laryngology, may cause problems for the surgeon, especially if he or she is not prepared, and for the patient with the risk of laryngeal injury and unachieved procedure. It is important to predict this situation to inform patients of DLE risks prior to surgery and to allow the surgeon to prepare by adding different tools, such as curved instruments and endoscopes, to his or her standard setting. Therefore, we conducted this prospective study to establish reproducible measures that can predict DLE.

The term DLE is still not well defined in the literature. Roh et al.^12^ proposed a classification for laryngeal view as follows: grade 1: full view of the vocal folds; grade 2A: partial view of the vocal folds, but the anterior commissure is not seen; grade 2B: partial view of the vocal folds (less than half); grade 3: only the arytenoids are visible; and grade 4: the entire glottis and arytenoids are hidden. They defined grades 3 and 4 as DLE. Piazza et al.^13^ defined DLE as visualization of the anterior commissure by a small-bore laryngoscope in the sniffing position and external compression or the impossibility of visualization of the anterior commissure and limitation of exposure to the posterior third of the vocal cord. Hekiert et al.^14^ proposed a visual analog score (VAS) of 1 to 10 assessed by the laryngologist, with 1 being the least difficult and 10 being the most difficult. However, although this definition varies according to the authors, the majority agree that exposure limited to the posterior third or less of the vocal cord defines DLE^9,12,13,15^. We applied the protocol proposed by Hsiung, which consists of evaluating laryngeal exposure after putting the patient in the sniffing position, external counterpressure and changing to a smaller laryngoscope if the exposure is not complete^9^. Therefore, DLE was defined in this study as exposure of the larynx limited to the posterior third of the vocal cord after the abovementioned efforts^9^.

Factors leading to difficult intubation are intricately related to those leading to difficult exposure during suspension laryngoscopy. Thus, it is usually believed that patients who are difficult to intubate are considered likely DLE candidates^8,12,15^. Likewise, a high Mallampati grade can predict high Cormack–Lehane scores and, consequently, DLE^8,9,15^. This was confirmed in this study since we found a strong correlation between high Mallampati and Cormack scores and DLE.

Since these scores depend on anatomic factors such as obesity, restricted mouth opening, macroglossia, and retrognathia, we chose to evaluate these factors to discover those that are independent predictors for DLE. Therefore, we measured the opening of the mouth to evaluate restricted mouth opening as well as the HMD, DTMD, VTMD, HTMD and SMD to evaluate retrognatia and the neck length, neck circumference, weight and BMI and thereby assess the impact of obesity and, especially, an excess of tissue located in the cervical region upon laryngeal exposure. We used all these measurements not only in the neutral position but also in the full extension head position to optimize other parameters because we believe that the cervical extension capacity has an important role in predicting DLE. Our results showed that limited mouth opening (< 49 mm), DTMD < 67 mm in the neutral position, DTMD < 82 mm and SMD < 157 mm at full extension of the head were statistically related to DLE in the univariate analysis. In the multivariate study, only the DTMD in the neutral position seemed to be correlated with the DLE (p: 0.049) when controlling the HTMD at full extension.

In fact, the results in the literature review of the impact of these physical measurements are variable, and their predictability has been questioned.

In their prospective study including 93 patients who underwent suspension laryngoscopy with 22 cases of DLE, Pinar et al. found that mouth opening was not correlated with DLE^8^. In another prospective study including 73 patients with 13 cases of DLE, this correlation was not found^12^. Some authors suggest that a full opening mouth is a good predictor for difficult intubation but not for DLE^12,16^.

In a multivariate study, Roh et al. found that DTMD < 55 mm and HTMD < 40 mm in a neutral position are correlated with DLE and concluded that retrognathia predicts DLE^12^. For Pinar et al., this correlation was found with measurement of the HMD and SMD in the full extension position, which are related to the degree of retrognathia and length of the neck^8^. However, in other studies, those measures were not good predictors for DLE^9,15^.

According to our results, DTMD seems to become correlated with DLE when controlling the HTMD. This means that for patients without retrognathia, the risk of DLE is higher in patients with shorter necks. In fact, the HTMD is more specific for the evaluation of retrognathia. Therefore, when the HTMD is the same, the DTMD will depend essentially on the length of the neck.

The statistical analysis of the radiographic parameters demonstrated that the effective length of the maxilla and atlanto-occipital distance were independent predictors for DLE (p: 0.015 and 0.001, respectively). In fact, the effective length of the maxilla was significantly larger in the DLE group. The increase in maxillary length reflects overgrowth of the maxilla and results in protruding upper teeth^17,18^, which is often cited as a cause of difficult direct laryngoscopy^18^. We believe that this measurement reflects not only an increase in the length of the maxilla but also an increase in the mass of its contents. Therefore, an increase in the maxillary length may increase the length, thickness and overall surface area of the soft palate and increase the tongue area.

The atlanto-occipital distance was significantly lower in the DLE group than in the control group, with a median of 4 mm and 6.5 mm, respectively, for both groups. A threshold value of 6 mm was fixed based on the defined cutoff values. This distance has been largely used to predict difficult intubation and measured, especially in neutral positions^17^, and it reflects the cervical extension capacity. Limited extension is correlated with difficulty in laryngeal exposure. This finding was confirmed in this study, in which the atlanto-occipital distance was significantly shorter in the DLE group than in the control group. Several authors^17,19^ reported a wide variation in the atlanto-occipital distance between DLE patients and non-DLE patients, and this parameter was considered a major predictive factor of difficult laryngoscopy. Nichol et al.^19^ demonstrated that a decrease in the atlanto-occipital gap or even contact of the posterior tubercle of the atlas with the occiput could be observed on both neutral and head-extended lateral cervical X-rays and could impair laryngeal exposure.

Kikkawa et al.^10^ found that the atlanto-occipital distance, which was measured on radiographic film performed with the head extended, was lower in the DLE group, with a median of 0.068 ± 0.15 versus 0.114 ± 0.22 in the control group, although the difference between the groups was not significant.

Piazza et al.^13^ analyzed the degree of neck flexion–extension by asking the patient to extend the neck and then measuring the arc from this position to that of full flexion of the neck onto the chest. They found that a flexion–extension degree less than 90° was significantly correlated with DLE. Paul et al.^15^ measured the atlanto-occipital extension degree and found that it was a good predictor of DLE if less than 19.5° (p: 0.001).

Other factors, including the presence of mandibular tori, a history of radiation, etc., can lead to DLE.

The main limitation of the present study was that the most reliable parameters for difficult laryngeal exposure were based on radiographic measurements, which imply ordering lateral cervical X-rays for every patient who will undergo phonosurgery. This examination, even though not time-consuming or invasive, constitutes an additional source of radiation exposure for the patient. However, we believe that X-ray assessment could effectively predict the risk of difficult laryngeal exposure; therefore, the surgeon could prepare for this event and prevent laryngeal trauma, incomplete resection or even abortion of the procedure by using different modalities, such as 30° and 70° endoscopes and angular surgical instruments, especially designed for this situation^2^, as well as flexible fibroscopy with two surgeons, as described by Kuang-Chih and Chih-Shin^20^. Therefore, we believe that allowing the surgeon to perform phonosurgery in the best conditions greatly outweighs the disadvantages of ordering a radiographic X-ray. We are also fully aware that not all hospitals worldwide are equipped with a standard X-ray machine, even though it is considered basic radiological equipment. We advise, in these cases, to reserve this imaging at least for patients who have clinical predictive factors for possible difficult laryngeal exposure, especially in cases of an anterior lesion (of the anterior commissure and/or the anterior 1/3 of the vocal cords). In these cases, imaging can be performed at the nearest facility equipped with this machine.

## Conclusion

In this study, several clinical parameters, such as mouth opening and the direct and horizontal thyromental distance, especially in the hyperextension head position, as well as the Mallampati and Cormack scores, were found to be good predictors of difficult laryngeal exposure. However, they were not found to be independent predictive factors in the multivariate analysis. Only two radiographic measurements (the maxillary length and the atlanto-occipital distance) were found to be significant independent predictive factors preoperatively for the occurrence of difficult laryngeal exposure during the microlaryngoscopy procedure. These parameters highlight the effect of overgrowth of the maxilla, protrusion of the upper teeth and limited extension of the cervical spine as major risk factors for difficult laryngeal exposure. Therefore, we recommend preoperatively ordering a lateral cervical X-ray with extension of the head and checking for these two radiographic measurements. Based on the current study, a maxillary distance greater than 90 mm and an atlanto-occipital distance less than 6 mm may indicate difficult laryngeal exposure.

## Data Availability

The datasets used and/or analyzed during the current study available from the corresponding author on reasonable request.
